# Determination of Environmental Pollutants

**DOI:** 10.1155/2013/750259

**Published:** 2013-04-07

**Authors:** Núria Fontanals, Jing-Fu Liu, Arunchalam Manimekalan, Sayan Bhattacharya

**Affiliations:** ^1^Department of Analytical and Organic Chemistry, Universitat Rovira i Virgili, Campus Sescelades, Marcel*·*lí Domingo, s/n, 43007 Tarragona, Spain; ^2^State Key Laboratory of Environmental Chemistry and Ecotoxicology, Research Center for Eco-Environmental Sciences, Chinese Academy of Sciences, Beijing, China; ^3^Biodiversity & Aquatic Ecology Laboratory, Bharathiar University, Coimbatore, India; ^4^Department of Environmental Science, Asutosh College, Kolkata, India

Over the last decades, there has been increasing global concern about the sources, sinks, distributions, fates, and effects of environmental pollutants. The ubiquitous presence in the environment and the adverse effects on environment, biotic community, and human health of these pollutants make it necessary to develop analytical methods to identify them and their degradation products as well as to quantify them at low concentration levels. 

Environmental chemistry in combination with analytical methods is a highly multidisciplinary science and covers many aspects that include (i) monitoring and identifying pollutants and their degradation/metabolic products at the low concentration levels in air, water, sediments, and biota; (ii) studying the toxicological effects of these pollutants in biota and human health; (iii) wasting utilization and management, among many others.

In this scenario this special issue of The Scientific World Journal is focused on determination of pollutants in all different environmental media. This topic is quite complex and broad as it is reflected by the diversity of the topic covered related to environmental analytical chemistry in the contributions.

As pointed before, quantification of pollutants in different environmental media is required to evaluate their fate. Two studies evaluate the presence of different families of pollutants in environment. The monitorization of organochlorine pesticides (OCPs) and metals in surface waters from the main basin in Turkey (M. E. Aydin et al.) and of volatile organic compounds (VOCs) in air from an industrial area in the Southern Italy (M. Amodio et al.) are two examples. Furthermore, two studies are focused on the investigation of models to predict the distribution of dissolved and particulate forms of 49 metals along 17 sites in Tigris River in Turkey (S. H. Hamad et al.) or to predict the dynamic correlation of air pollution indexes (APIs) between 42 cities in China (Z. He et al.). Finally, another study (U. Mutman) presents a strategy based on the use of biomass to reduce waste, specifically, the reuse of olive oil waste ash as clay stabilizer and, in this way, close the gap in environmental field.

